# Patient perspectives on delays in care for kidney stones: A qualitative analysis

**DOI:** 10.1371/journal.pone.0341787

**Published:** 2026-06-01

**Authors:** Ibukunoluwa I. Ibrahim, Daniel L. Zager, Leslie B. Charondo, Sophia Zamudio-Haas, Sara L. Ackerman, David B. Bayne

**Affiliations:** 1 School of Medicine, University of California, San Francisco, California, United States of America; 2 Department of Urology, University of California, Davis, California, United States of America; 3 Department of Medicine, University of California, San Francisco, California, United States of America; 4 Department of Social and Behavioral Science, University of California, San Francisco, California, United States of America; 5 Department of Urology, University of California, San Francisco, California, United States of America; China Medical University, TAIWAN

## Abstract

**Purpose:**

Traditional insurance claims dataset analyses have exposed disparities in treatment delays for kidney stones along lines of socio-economic status, particularly among patients who are underinsured and/or racial/ethnic minorities. Analyzing patient experiences through qualitative semi-structured interviews allows for elucidation of common root causes leading to care delays in patients with kidney stones.

**Materials and methods:**

20 participants were recruited from a group of adult patients at a safety net hospital and academic medical center who had been referred for urological care after presenting to the Emergency Department for kidney stones. Patients were selected for semi-structured interviews if they failed to present to the urology clinic after 60 days of referral placement. Interviews were conducted in Spanish or English. Transcripts were coded and analyzed using thematic analysis.

**Results:**

Of the 20 participants, the median age in years was 46 (range 22–72), 50% identified as female, 40% were White, 40% were Hispanic, 20% were Black, and 20% were Spanish-dominant. The median delay was 105 days. Thematic analysis identified patient-extrinsic obstacles in obtaining timely care in the form of care costs and skepticism from health care providers, such as perceptions of being drug-seeking or feigning pain. We further identified patient-intrinsic obstacles, such as gaps in understanding of the dangers of stone disease and difficulty navigating care coordination.

**Conclusions:**

This study provides insights into multi-level factors that impact care delays for patients with kidney stone disease. Findings from this work deepen understanding of why patient experiences in care delays manifest along lines of socio-economic status and can inform future interventions to reduce disparities.

## Introduction

Kidney stones affect an estimated 9.9% of people in the United States, and their prevalence is increasing [1,2]. Close to half of all patients who present to the Emergency Department (ED) for kidney stones do not receive any follow-up care [3]. Lack of follow-up has significant health consequences. Delayed treatment for stones is associated with increased need for complex surgical care and places patients at increased risk of renal damage, urosepsis, pain, and death downstream of untreated stone disease [4–8]. While all patients with kidney stones report a decrease in quality of life when living with stones [9], this is more pronounced among patients who are ethnic minorities and lower income [10], who are more likely to experience delays in their treatment and harm from these delays [11].

Despite documented disparities in quality of life, timely access to care, and surgical morbidity among vulnerable patient populations, there is a persistent gap in our understanding of the individual and interpersonal factors that drive these disparities. While studies have identified that patients report an interest in learning more about stone prevention and strong motivation to reduce stone recurrence [12,13], there is a need to better understand the underlying barriers that limit patients’ capacity to advocate and obtain care for their stone disease. This inquiry is an essential step to developing clinical interventions that empower patients and providers to prevent and reduce inequities in kidney stone care.

The research reported here aimed to elucidate patient perspectives on the root causes or contributors to delays in treatment for their kidney stone disease. Drawing on a real-world sample of patients who presented in emergency settings with kidney stones and experienced delayed follow up, we conducted qualitative research to understand common pitfalls patients face and potential opportunities for reducing care delays from ED presentation to urology clinic visit. Increased understanding of these challenges provides direction for future strategies to empower patients and providers to improve care and reduce delays in the treatment of kidney stones.

## Materials and methods

### Research design

We interviewed adult patients who were referred for urological care after presenting to the ED for kidney stones at hospitals within the University of California, San Francisco (UCSF) health system and failed to follow-up in the urology clinic 60 days after referral placement. Interviews offered the opportunity to learn about patients’ experiences seeking care for kidney stones, understanding and expectations of the referral process, and thoughts and feelings about interacting with healthcare providers [14,15].

### Sampling strategy, recruitment, and ethics

We conducted a chart review of patient records in the electronic record referral queue at Zuckerberg San Francisco General Hospital (SFGH), a safety net hospital, and UCSF Medical Center, an academic medical center, over the six-month period between July 1st, 2023 and January 1st, 2024 to identify participants for recruitment. Acknowledging that no consensus exists on the definition of safety net setting, this study based it largely off the Institute of Medicine definition of a hospital that delivers care to a large share of medically and socially vulnerable patients irrespective of their ability to pay‌‌ [16]. III and DLZ contacted individuals via phone call. Participants received a $60 visa gift card. This study was approved by the UCSF institutional review board (IRB #21–35933). We obtained written consent from all participants. All procedures performed in this study involving human participants were in accordance with the ethical standards of the UCSF Internal Review Board and with the 1964 Helskini Declaration and its later amendments.

### Data collection

Authors DBB and SLA developed a semi-structured interview guide (S1 Text) to explore the following domains: patients’ initial symptoms of presentation; experiences with providers and staff; knowledge about their conditions; support from loved ones; and impact of diagnosis on personal, social and financial well-being. Questions asked about patients’ experiences communicating with provider and emotional reactions to treatment. Prompts encouraged reflection on challenges encountered while seeking care, as well as support received.

Authors DBB, III, and DLZ conducted interviews and collected demographic information in participants’ preferred language, over phone, zoom, or in-person, depending on participant preference. Quotes originally in Spanish were translated to English; S1 Table provides the original Spanish quotes. Authors DBB, III, DLZ, and LBC reviewed transcripts and provided feedback on technique. We audio-recorded, transcribed, de-identified, and reviewed for accuracy.

### Data processing and analysis

We used Dedoose to code and organize data (Version 9.2.12). Authors DBB, DLZ, III and LBC individually coded five transcripts, discussed preliminary codes, and created a final codebook (S2 Table). All transcripts were double coded by authors DBB, DLZ, III, and LBC, who compared coding and resolved any inconsistencies. We generated a heatmap to identify recurrent cross-codes (S1 Fig). III, DLZ, LBC, and DBB conducted concurrent data collection and analysis, until we determined we reached saturation of themes and conceptual depth, after 15 interviews [17–19]. This determination was completed in accordance to qualitative standards to ensure redundancy of previous data within transcripts and lack of emergence of new data [19]. We completed five additional interviews to ensure saturation.

### Reflexivity and research team description

Reflexivity was considered and discussed [20]. Interviewers DLZ and III were first- and second-year medical students, respectively, with limited urology expertise, facilitating an unbiased explorative approach to urological care. This potentially increased patients’ comfort sharing personal experience, without fear of repercussions. Offering interviews with native Spanish speaker DLZ, may have enhanced rapport and supported authentic expression. Other investigators included a urology resident (LBC) with significant experience conducting qualitative research, a urologist with a strong research background in clinical, social, and behavioral factors that contribute to stone disease (DBB), an anthropologist with expertise in qualitative research methods (SLA), and a public health professional with qualitative research and technical writing experience (SZH).

## Results

Patient characteristics are summarized in Table 1. The time between ED and urology visits was a median of the 14 participants who completed follow-up within 365 days. No patient required emergent drainage or experienced sepsis. We identified three themes related to delays in care. The first theme evoked the substantial financial burden patients incurred related to stone disease. The second described pain and how patients felt their pain was perceived by providers. The third exposed failures to educate patients in health literacy and navigation of the healthcare system. Interviews highlighted that advocacy was often necessary to advance care. Additional quotes supporting themes were listed in Table 2.

**Table 1:** Characteristics at time of each interview.

Characteristic	N (%)	Median (IQR)
Age, in years		46 (21.5)
Gender		
*Female*	10 (50%)	
*Male*	9 (45%)	
*Nonbinary*	1 (5%)	
Race/Ethnicity		
*Black*	4 (20%)	
*Hispanic/Latino*	8 (40%)	
*White, Non-Hispanic*	8 (40%)	
Primary language		
*English*	16 (80%)	
*Spanish*	4 (20%)	
Insurance		
*Public*	16 (80%)	
*Private*	3 (15%)	
*None*	1 (5%)	
Hospital setting		
*Safety net*	17 (85%)	
*Tertiary academic center*	3 (15%)	
Time from ED visit to urology visit, in days*		99 (129)^a^
Stone size, in mm		4 (2.5)
Presence of obstructing stone		
*Yes*	15 (75%)	
*No*	5 (25%)	
Interview duration, in minutes		21 (13)

^a^Six patients who had greater than 365 days to follow up were included in the study but were not included in this distribution.

**Table 2:** Additional quotes organized by theme.

Theme	Quotes
The Financial Burden of Medical Care	Patient C: I know that [my insurance] is going to be ending soon. Next year so if this were to happen a year later. I would have definitely had a financial impact where I might not even have gone to the hospital to be honest just because I know that money is tight and I probably wouldn’t have my current benefits.
	Patient J: I was not able to work. And my initial appointment got cancelled and I had already taken off work. It just put me in limbo for a month.
	Patient O: I went to the emergency room. I went there because I didn’t know I had insurance.
	Patient P: They shouldn’t charge me an arm and a leg… I just feel like people shouldn’t be charged just to get their health together. You know what I mean? People deserve life.
Perceptions of Pain: Patients’ Experience of Bias in their Care	Patient E: I told them I could not manage the level of pain…They do not see blood or broken bones, so they think in the absence of these injuries, there must not be such intense pain.^a^
	Patient G: Quite often as [a] patient, in order to get the attention of medical professionals––who is also being told by drug addicts that their pain is 1 billion on the scale of one to 10––you kind of have to talk it up a bit. Which I haven’t yet.
	Patient H: I was a wreck. I was begging for help. And she [nurse] was trying to comfort me but I waited a really long time to be seen by a doctor and get out of the pain. I was in acute pain.
	Patient A: Me asking for some pain medicine made me feel like they didn’t want to give it to me, because the crisis in [the city] is really high of opiate use…Here’s a patient that never asked for any pain medicine, but still I was treated as if I was part of that opiate pandemic… I was told, oh, well, you gotta see a pain specialist… Once they found that stone, you felt like, okay, well, now you can believe me, now you’re listening more to what I’m saying.
Failure in Patient Education	Patient I: His bedside manner, in the initial visit was okay. I think I saw another doctor for the results and he was just kind of to the point and cold… And he didn’t provide any additional information. Nothing, apart from the results.
	Patient R: I had this appointment on my MyChart and I did not know what this appointment was for, So I didn’t go, and then they call me the next Monday, and they told me that I missed the appointment.
	Patient D: I can’t remember if I was ever told how [the stones would be removed]. It still feels like, I don’t have anything
Advocacy	Patient A: Before me going into [the hospital] as my primary I had an outpatient clinic like a little small clinic that had my primary there and that primary physician retired during the pandemic. So it took him a while to get me in and to be seen. I had to contact [my insurance]...It took me a while to get the correct health care in which I’m getting right now.
	Patient K: I contacted my family doctor….And I asked to set up an appointment with [a] urologist. And also to do [imaging] of all my kidney and so on, so I can see what is going on there.
	Patient G: Although getting a hold of on the telephone is a pain...Exchanging messages back and forth to get stuff delivered…I’m reasonably knowledgeable about how to get these systems to work.

^a^Originally in Spanish and translated to English.

### The financial burdens of medical care

Patients incurred substantial financial burden due to their disease. Across interviews, participants emphasized direct costs (e.g., medication, hospital bills) and indirect costs (e.g., wages lost, transportation, relationships impacted) and expressed significant concerns surrounding these costs, including stress due to missed work for ED visits or follow-up appointments. Participants described how financial factors resulted in self-deferral of treatment.

[After my emergency department visit], I made an appointment with the urologist, but then I got a bill for $20,000. So yes, I got scared…I’m waiting to go to [my country] to get treated there. Because that is too much money, and I don’t have the resources to pay that. (Patient B, translated from Spanish)

In instances where safety net insurance coverage from the county hospital ameliorated the financial burden, loss of wages and other indirect costs of care still impeded patients’ ability to seek treatment with the urologist.

The hardest part was trying to communicate to my work…I’m a contractor and not afforded sick days…So, if I don’t work, I don’t get paid…I was kind of forced to work, because this kidney stone episode was taking close to two months to pass. (Patient N)

### Perceptions of pain: patients’ experience of bias in their care

Anticipated and experienced bias hindered patients seeking care. Patients frequently feared that providers would have a negative perception of them prior to an encounter, shared experiences of symptoms not taken seriously, or having to strongly advocate to receive care, particularly in the ED. Patients required repeat visits to receive the correct diagnosis. Patients described being doubted, as though they were seeking care for secondary gain, such as pain medication. Participants described being met with scrutiny and skepticism from staff, due to repeated presentations.

They weren’t taking my pain seriously, and they thought I was playing until they saw me throw up. (Patient Q)

I think they thought I was exaggerating about the pain and that I was being dramatic... Or that I was like there for like pain meds or something because I wasn’t treated very well. (Patient N)

### Failure in patient education

Failures in patient education, reflected in poor health literacy and ability to navigate the healthcare system, proved a barrier to care access and treatment progression. Patients described feeling uneasy about being discharged with little explanation of their condition, with minimal direction or plan.

It was very confusing and I can’t really tell you this is what the kidney stone do[es] and this is how it[‘s] affecting my body. So, yeah, just a better way to actually stay on the same page on my diagnosis would’ve been great. (Patient C)

I had to look up how to drink more water and what foods to avoid. Maybe if they told me that would have been helpful. I know I can do that stuff on Google, but it would have been nice to have it [from the doctor]. (Patient R)

Patients also experienced failures in education regarding how to navigate the referral process to urology. Gaps in communication compounded by long wait times caused significant negative experiences for patients that they perceived as inefficient and error ridden.

I think that it would have been great if the ER could have done a referral for me to a urologist just to expedite that process. (Patient N)

There was no real aftercare…It would be nice to get a phone call or just more aftercare to see if I am okay…It leaves it on myself, as the patient, to really investigate and reach out to my doctor. (Patient C)

### Advocacy as a critical tool to garner better care

**Fig 1:**
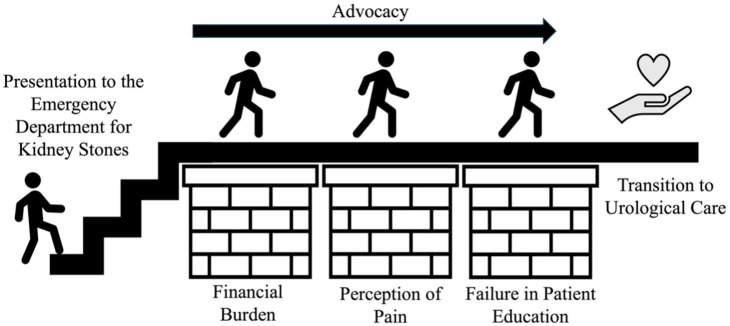
Conceptual model of patient advocacy and barriers contributing to delays in kidney stone care. The diagram summarizes key themes (financial burden, perceptions of pain-related bias, and failures in health literacy) and illustrates how advocacy can mitigate barriers to timely follow-up.

Advocacy emerged as a theme in the advancement of patient care for kidney stones. Fig 1 illustrates this relationship between advocacy and barriers. Patients described self-advocacy, advocacy from their support network of family and friends, and advocacy empowered by healthcare workers who took the time to teach patients about the urgency of their situation. In the quote below, a participant shared how she advocated to receive diagnosis.

The doctor told me that based on the exams they did, there were no kidney stones…I returned several days later still in a lot of pain, and the same doctor was present, and I told him, ‘It’s just I still feel sick. But this time, I am not leaving here until you tell me whether I have kidney stones…I would like to get some imaging.’ After that, he ordered the imaging, and he told me, ‘You were right, these are kidney stones.’ So, it is important to listen to the patient. (Patient E, translated from Spanish)

Family advocacy in the form of emotional and financial support was vital for patients in the advancement of their care. In interviews, patients shared it was common practice for their loved ones to be active participants during their care.

My daughter accompanied me to the Emergency Room, [my whole family was involved with my care]…[They] brought me food and that is why I did not spend much money …My daughter was giving me instructions on how to take [medication]. (Patient M, translated from Spanish)

While many patients left their interactions with providers in the ED feeling confused about their diagnosis and next steps, some patients shared they felt that they had strong emotional connections and positive experiences with providers.

I mean, I feel good about seeing them, that’s one step closer to finding out what’s going on, what’s going to happen…I’m pretty sure that the urologist knows what he or she is doing. It’s a good thing to see the doctor because…[the] doctor knows about certain parts of the body that I don’t...I feel good. (Patient P)

Even though patients were in pain, had long wait times, and were worried at presentation, emotional connection with clinicians and a sense of trust helped bring relief when receiving needed care.

## Discussion

This formative study leveraged patients’ perspectives to describe the central barriers that created delays in kidney stone treatment, across the cascade of care. Previous qualitative studies have focused on changes in patients’ quality of life, but to our knowledge, this is the first to assess reasons for delays in treatment [9,21,22]. We identified three primary themes that explained why patients from disadvantaged backgrounds, many of whom had limited English, experienced delays: financial burden, provider bias in care, and a system level failure to educate patients about their condition and how to navigate follow-up.

Findings from our thematic analysis of in-depth interviews contextualized previous quantitative studies on delays in kidney stone treatment and bias in ED settings. Participants identified cost as the most important factor they considered when deciding to seek care and access specialists. Patients who shared concerns about insurance coverage avoided seeking care due to fear of large medical bills, while patients with insurance still reported concerns about missing work and losing income as a result. These narratives expand on previous quantitative research showing that underinsured patients were more likely to experience delays in care [11] and is congruent with quantitative research demonstrating that one third of adults treated for nephrolithiasis missed work for their condition [23].

Anticipated and experienced provider bias discouraged patients with kidney stones during their care process. If patients felt their symptom descriptions were perceived as malingering or as having ulterior motives, their confidence in providers was eroded and their pursuit of care was discouraged [24,25]. While bias in pain management in the ED setting for disadvantaged populations and ethnic and racial minorities has been documented [26,27], results from this study uniquely documented how this might contribute to kidney stone care delays and point to an opportunity for continued provider education. A previous study in stone care suggests that patients have improved satisfaction when provided with educational materials [28].

We found that insufficient health communication to participants about their diagnoses and the procedures needed for follow-up led to delays in care. Lack of appropriate and accessible information for patients limited their health literacy [29], including knowledge around how to navigate care advancement. Previous studies have documented that limited health literacy among patients is associated with difficulties in medical management of chronic conditions, decreased medication adherence, and difficulties in communication with providers [30,31]. A quantitative study in the same geographic area with similar patient population as our study also found that patients with limited health literacy experienced increased effort and frustration when seeking health-related information [32]. Our findings added to this and suggest an opportunity for improved communication for follow-up care.

Our study demonstrated the power of a positive patient-provider connection and the importance of trust in kidney stone care. Strong patient-provider therapeutic alliance has been shown to improve outcomes for other conditions, and can potentially be applied to stone disease care [20]. Care advocates and navigators have been proven to improve outcomes for marginalized patient populations [33,34]. Findings from our interviews provide the foundation for future research directions to develop, pilot and test peer navigation and advocacy interventions to improve care follow up for kidney stone disease.

This study has a few important limitations. Most participants were from a safety net setting, potentially limiting the applicability of findings to other contexts. The two hospitals represented in this study serve a diverse urban population. No participants were of Asian, Native American, or Pacific Islander descent, and thus missed the perspectives unique to these groups. This study may thus be missing perspectives unique to these non-represented ethnic groups or patients in non-urban settings. In future studies, it will be important to include the perspectives of care providers or family members, given the role they play as advocates for stone disease care.

## Conclusions

Our study provided important and actional able insights into patient perspectives on barriers to receiving care for kidney stones. This study has to be interpreted with caution because it only is composed of perspectives of 20 predominantly safety net hospital patients in a single region of one state. However, it does expose previously not described experiences of kidney stone patient who face care delays. We believe it is important to more broadly investigate patient perspectives and opportunities for advocacy as a potential solution in other stone patients. Here, we documented examples of how patients can overcome barriers through self-advocacy, or advocacy in the form of social support. This points to the potential for patient support interventions through advocates to reduce care delays for socially at-risk patients with kidney stones.

## Supporting information

S1 Text Semi-structured interview guide. Interview guide used for patient interviews.
(DOCX)

S1 Table Original Spanish quotes. Original Spanish-language quotes corresponding to translated quotations in the manuscript.
(DOCX)

S2 Table Final codebook. Codebook used for thematic coding and analysis.
(DOCX)

S1 Fig Heatmap of recurrent cross-codes. Heatmap depicting recurrent cross-codes across interviews.
(PNG)
