# Supplementary material for: Patient perspectives on delays in care for kidney stones: A qualitative analysis
Source: PLoS One. 2026 Jun 1;21(6):e0341787. doi: 10.1371/journal.pone.0341787 (PMC13225416; doi:10.1371/journal.pone.0341787)
Supplement: S1 Text — Interview guide used for patient interviews. (DOCX) [file pone.0341787.s001.docx]

**Supplementary Text 1.** Interview guide.

Hello, how are you?

We are conducting interviews to try to figure out how we can do a better job taking care of patients with kidney stones. Our conversation will last about an hour. There are no ‘right’ or ‘wrong’ answers to our questions. We are interested in your own perspective. If there are any questions that you don’t feel comfortable answering, just let us know and we will move on. With your permission this interview will be recorded and transcribed. The recording and transcript will be held securely by the research team. Transcripts will be anonymized, which means that all names and other identifying information will be removed, will not be shared beyond the research team, and will not be included in reports or publications. You are free to not answer any questions or stop the interview if you wish. Before we begin I just want to make sure you are not in severe pain or on any strong pain medications. Please let me know if this is the case and we can reschedule our interview. Do you have any questions before we begin? Do I have your permission to begin recording?

1. Can you tell me what your symptoms were like before your stone was found/diagnosed?
2. Tell me about your decision to seek out care for your symptoms. [Probe for where care was sought and whether/why care seeking was delayed, including financial barriers.]
3. What was it like when you first saw a doctor about your condition?
4. What did the doctor tell you about your condition? [Probe for experience receiving a diagnosis, understanding of diagnosis, and perceived severity of condition.]
5. After you saw the doctor and learned that you had a stone, how did you feel?
6. Did you talk to your family or friends about your diagnosis? [Probe for nature of conversations and whether they spoke with others who have stones.]
7. What worries you most about having kidney stones, if anything?
8. After you got your diagnosis, what happened next with your care? [Probe for whether anyone explained the treatment plan.]
9. What was difficult for you during this whole process?
10. How could the hospital or your doctor be doing a better job to make sure you get treatment for your stones?
11. How do you feel about seeing the urologist?
12. Did you ever think about seeing a different urologist than the one you were initially referred to?
13. What was this experience like for you financially, how did it affect you financially?
14. How did/does your stone disease impact relationships in your life? [Probe for interpersonal relationships, community relationships, societal relationships.]
15. What have you heard about passing kidney stones naturally? [Probe for whether this knowledge affected decisions.]
16. Did anyone discuss with you what you needed to do to prevent stones?
17. Is there anything about your experience with kidney stone care that I didn’t ask but should have?
