# Supplementary material for: Patient perspectives on delays in care for kidney stones: A qualitative analysis
Source: PLoS One. 2026 Jun 1;21(6):e0341787. doi: 10.1371/journal.pone.0341787 (PMC13225416; doi:10.1371/journal.pone.0341787)
Supplement: S1 Table — Original Spanish-language quotes corresponding to translated quotations in the manuscript. (DOCX) [file pone.0341787.s002.docx]

**Supplementary Material**

**Supplementary Table 1.** Original Spanish text of quotations translated into English.

| **English** | **Spanish** |
| --- | --- |
| Patient B:  [After my emergency department visit], I made an appointment with the urologist, but then I got a bill for $20,000. So yes, I got scared… I’m waiting to go to Mexico to get treated there. Because that is too much money, and I don’t have the resources to pay that. | Patient B:  [Después de mi visita a la sala de emergencias,] hice una cita para el urólogo pero ya después me llegó un recibo de 20 mil dólares. Entonces pues sí, me asuste…Así estoy esperando ir a México y para allí hacerme el tratamiento. Y porque la verdad, sí, es demasiado dinero y no tengo cómo pagar eso. |
| Patient E:  I told them I could not manage the level of pain…They do not see blood or broken bones, so they think in the absence of these injuries, there must not be such intense pain. | Patient E:  No podía aguantar [el dolor]…Si no ven sangre, no ven huesos quebrados, ósea piensan que cuando no están viendo en ese momento, no puede producir un dolor intenso. |
| Patient M:  My daughter accompanied me to the Emergency Room, [my whole family was involved with my care]…[They] brought me food and that is why I did not spend much money [during my recovery]…My daughter was giving me instructions on how to take [medication]. | Patient M:  Mi hija me acompañó a la emergencia y mi hija me decía, ‘yo la veo muy mal.’… Sí, me lo comenté [a mi familia de mi diagnosis], porque tengo algunas hermanas viviendo aquí en California. Ya como si mi hija se dio cuenta ya todos saben, ‘está en el hospital ella, mi mamá qué les pasó.’ Y ya empiezan a preguntar ya todas se dieron cuenta en el mismo rato casi. Y estaban así bien preocupadas…  Sí ellas me llevaban la comida y entonces por eso creo razón creo que no gasté mucho dinero [durante mi recuperación]…  [Mi hija] me estaba dando las instrucciones [sobre los medicamentos] así se los tiene que tomar. |
| Patient E:  The doctor told me that based on the exams they did, there were no kidney stones…I returned several days later still in a lot of pain, and the same doctor was present, and he said, ‘What are you doing here again ma’am?’ I told him, ‘It’s just I still feel sick. But this time, I am not leaving here until you tell me whether I have kidney stones…I would like to get some imaging.’ After that, he ordered the imaging, and afterward, he told me, ‘You were right, these are kidney stones.’ So it is important to listen to the patient. | Patient E:  El doctor me dijo que según los exámenes que hicieron, no eran cálculos.…Al otro día regresé muy adolorido, y el mismo doctor estaba y él decía, “¿Usted que se hace aquí otra vez señora?” Le digo, “es que sigo mal. Pero esta vez no me voy de aquí hasta que me dicen si tengo cálculos…Entonces quiero hacerme un examen.” De ahí es que el ordenó el examen, y luego lo volví a ver ese mismo día, y ahí me dijo es que “si tiene razón, son cálculos.” Entonces es importante escuchar al paciente. |
