# Supplementary material for: Patient perspectives on delays in care for kidney stones: A qualitative analysis
Source: PLoS One. 2026 Jun 1;21(6):e0341787. doi: 10.1371/journal.pone.0341787 (PMC13225416; doi:10.1371/journal.pone.0341787)
Supplement: S2 Table — Codebook used for thematic coding and analysis. (DOCX) [file pone.0341787.s003.docx]

**Supplementary Table 2:** Final Codebook

| **Parent Code** | **Description** | **Child Code** | **Description** |
| --- | --- | --- | --- |
| Systematic barriers to referral or follow up | Barriers that result in a delay of care that are extrinsic to the patient | Insurance Coverage | Barriers brought on by insurance (e.g. coverage, cost/co-pays) |
|  |  | Care delivery failure (previously known as clinic workflow barriers) | Clinic workflow that may impede patient follow up (e.g. delay in reaching out to patient to schedule, no follow-up if patient is lost to follow-up, lack of follow-up, vague discharge instructions, failure to communicate treatment plan, retirement) |
|  |  | Limited specialist availability | Care after referral (e.g. limited options to follow up, inability to reach clinic or schedule follow-up) |
|  |  | Overburdened healthcare system | Hospital/healthcare system barriers (e.g. long wait, inability to schedule imaging/labs, lack of information at discharge) |
|  |  | Outside current events | Effects of environment contributing to access to care (e.g. Pandemic) |
| Patient barriers to initial visit and follow up | Barriers that result in a delay of care that are intrinsic to the patient or their circumstances | Digital literacy | Navigating the online medical record, sending message to provider, scheduling appointments and imaging |
|  |  | Health literacy | Understanding of medical condition and navigating the health care system (e.g. intrinsic knowledge around stones and treatment, prior stones in family/friends, stone prevention mgmt) |
|  |  | Language barrier | Lack of interpreter use, documents in English |
|  |  | Competing social responsibilities or comorbidities | Duties or responsibilities that may limit opportunities to seek care (e.g., work, children) |
|  |  | Lack of social resources | (E.g. Lack of transportation, lack of health insurance or stable income, lack of support from family, societal stigma) |
| Patient experiences | Patient perception or experience in their care | Initial visit/diagnosis | Patients’ experience at their initial presentation/interaction with the healthcare system (e.g. suitable vs. long wait times, treated with respect vs. disrespect) |
|  |  | Follow through | Negative or positive follow up to care (e.g. rapid follow-up after initial visit, or lack of continuity in follow up appointment) |
|  |  | Pain | Patient experience of pain or anticipated pain with kidney stones (e.g. severe symptoms that motivated a patient to seek care, or fear of painful treatments that may discourage seeking care) |
|  |  | Emotional connection | Emotional support from providers (e.g. symptoms taken seriously, sympathize with a provider who may be busy) |
|  |  | Patient evaluation of treatment  (previously: Perceived success/failure of treatment or management) | Treatment and management for stones that patients appreciated (e.g. medications such as pain meds or Flomax, surgery), or treatment did not improve patient’s symptoms or presentation. Also can include feedback from the patient and their desired outcomes of appointments or treatment |
|  |  | Support provided (extrinsic) (previously resources) | Patients feel well-supported and well-informed (e.g. educational and/or physical support from family, friends, Youtube videos, online, handouts provided by provider) |
|  |  | Provider and patient skepticism (trust) | Provider does not believe patients’ symptoms or take them seriously, and patient does not trust the provider (e.g. provider was unhelpful, vague, or unsupportive in patient’s view, patient seeking second opinion, patient uncomfortable with provider) |
|  |  | Emotional disconnect | Patients felt mistreated or that the healthcare team was rude |
