# Supplementary figures and images for: Patient perspectives on delays in care for kidney stones: A qualitative analysis
Source: PLoS One. 2026 Jun 1;21(6):e0341787. doi: 10.1371/journal.pone.0341787 (PMC13225416; doi:10.1371/journal.pone.0341787)

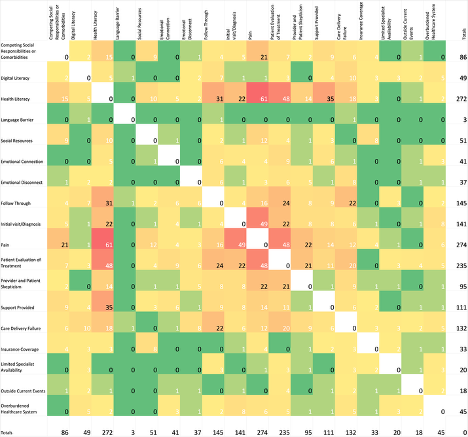

Supplement: S1 Fig — Heatmap depicting recurrent cross-codes across interviews. (PNG) [file pone.0341787.s004.png]
